# Puerarin Attenuates the Cytotoxicity Effects of Bisphenol S in HT22 Cells by Regulating the BDNF/TrkB/CREB Signaling Pathway

**DOI:** 10.3390/toxics13030162

**Published:** 2025-02-25

**Authors:** Meilin Qin, Xinxin Guo, Nuo Xu, Yan Su, Mengfen Pan, Zhengbao Zhang, Huaicai Zeng

**Affiliations:** 1 Guangxi Key Laboratory of Environmental Exposomics and Entire Lifecycle Health, School of Public Health, Guilin Medical University, Guilin 541199, China; qmeilin2022@163.com (M.Q.); guoxinxinw@126.com (X.G.); 19109628086@163.com (N.X.); dplyaya@163.com (Y.S.); panmfpan@163.com (M.P.); zhzhb12345@163.com (Z.Z.); 2 Guangxi Health Commission Key Laboratory of Entire Lifecycle Health and Care, School of Public Health, Guilin Medical University, Guilin 541199, China

**Keywords:** bisphenol S, puerarin, oxidative stress, synaptic plasticity, BDNF/TrkB/CREB signaling pathway

## Abstract

Bisphenol S (BPS) is a widespread environmental endocrine disrupter that can cause hepatotoxicity, neurotoxicity and negative effects on reproduction. Puerarin (PUE) has been found to have anti-inflammatory, antioxidant, and neuroprotective properties, however, its potential protective effects against BPS-induced neurotoxicity and the underlying mechanisms are still not fully understood. In this study, HT22 cells were exposed to different concentrations of BPS with or without PUE. Cell viability, apoptosis, oxidative damage, and the expression level of axon-injury-related genes and the BDNF/TrkB/CREB pathway were analyzed. The results showed that 40 μM to 180 μM BPS and 100 μM to 180 μM PUE significantly decreased the cell viability of HT22 cells, but in the 80 μM PUE group, the cell viability was higher than control group, and the ratio of 1.1. Meanwhile, BPS increased the production of ROS and MDA but decreased the GSH and SOD. However, supplementation with PUE was alleviated the oxidative damage. PUE also alleviated the apoptosis rate that induced by BPS. Additionally, BPS decreased the expression levels of mRNA and proteins of synaptic-related genes, but inhibited the expression levels of mRNA and proteins of the BDNF/TrkB/CREB signaling pathway. Interestingly, PUE was found to significantly recover the expression of synaptic related genes, but also upregulated the expression of the BDNF/TrkB/CREB pathway. In conclusion, our study proved that PUE can attenuate the neurotoxicity effect of bisphenol S, which related to oxidative damage in HT22 cells by regulating the BDNF/TrkB/CREB signaling pathway. This study is not only the first to demonstrate that PUE can mitigate BPS-induced neurotoxicity through oxidative stress modulation, but also provides a novel therapeutic approach involving the BDNF/TrkB/CREB pathway. These findings offer promising insights into natural-based strategies for protecting against environmental neurotoxins and provide a foundation for future therapeutic developments targeting BPS-induced neurotoxicity.

## 1. Introduction

Bisphenol S (BPS) is widely used as a safe alternative to Bisphenol A (BPA). In the European Economic Area, the annual manufacturing import volume of BPS exceeds 10,000 tons [1]. A study was conducted to the concentration of bisphenol analogs in indoor dust samples obtained from China, the United States, South Korea, and Japan, and the results showed that the concentrations ranged 0.00083 to 6.6 μg/g [2]. In addition, multiple studies have shown that the BPS concentration in water samples is nearly equivalent to that of BPA, with concentrations of between 0.1 and 10 ng/L [3,4]. Meanwhile, BPS has been widely detected in the food and human biological samples such as plasma and urine [1]. Hence, the increasing use of BPS is raising important public health concerns.

**Figure 1:**
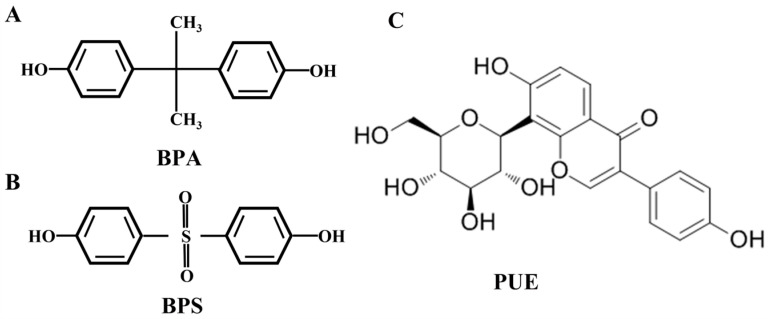
The chemical structures of BPA, BPS, and PUE. (**A**) BPA; (**B**) BPS; (**C**) PUE.

Compared with BPA, BPS has weaker estrogenic activity and a similar chemical structure to BPA (Figure 1) [5]. Jiang et al. were the first to provide population data on the effects of BPS on neurodevelopment, demonstrating that prenatal BPS exposure induces neurodevelopmental toxicity in offspring [6]. Our previous studies also demonstrated that BPS has negative effects on the reproductive system [7], and induces hepatotoxicity [8] and neurotoxicity effects [9] in vitro and in vivo. Studies using male IMR-32 and female SK–N–SH cell lines exposed to BPS for 24 h showed that BPS induced oxidative stress. This is achieved by promoting reactive oxygen species (ROS) production, increasing malondialdehyde (MDA) levels, and reducing superoxide dismutase (SOD) activity [10]. A study using an in vitro model of BPS treatment in rat adipose-derived stem cells and human mesenchymal stem cells showed that BPS exposure is associated with decreased cell viability and increased cell apoptosis [11]. Apart from inducing oxidative stress and apoptosis, BPS can reduce the number of synapses in the hippocampus and alter neurological functioning in mice [12]. Brain-derived neurotrophic factor (BDNF) is important for functioning and structural plasticity in the central nervous system. Furthermore, it exerts its effect by binding to tyrosine receptor kinase B (TrkB), thereby activating the cAMP response element-binding protein (CREB) [10,13]. Our previous studies found that BPS inhibited the BDNF/TrkB/CREB pathway in SK-N-SH cells and mice. However, synaptic plasticity can be reversed by activating the BDNF/TrkB/CREB signaling pathway [13,14]. Therefore, BPS may induce neurotoxicity through mechanisms such as neuronal apoptosis and oxidative stress, while inducing axonal injury by inhibiting the BDNF/TrkB/CREB signaling pathway.

Recently, many studies have posited that natural compounds may have the potential to alleviate the negative impacts of environmental toxins [15]. Puerarin (PUE), a primary bioactive component of Pueraria lobata, exhibits various pharmacological effects, such as anti-inflammatory, antioxidant, anti-apoptotic, and neuroprotective effects [16]. A clinical study showed that PUE could play a role in treating ischemic diseases by inhibiting oxidative stress [17]. Additionally, PUE regulates H_2_O_2_-induced cell growth restriction and significantly reverses H_2_O_2_-induced apoptosis [18]. PUE exerts neuroprotective effects by increasing the number of hippocampal neurons and improving neuroplasticity in mice. Furthermore, a study showed that PUE may improve neurological function through the upregulation of BDNF and TrkB expression [19]. PUE may play a neuroprotective role by reducing neuronal apoptosis, alleviating oxidative stress levels, and improving synaptic damage [20]. Our previous study showed that PUE could reduce the release of inflammatory factors and improve abnormal liver lipid metabolism induced by BPS exposure.

Therefore, we hypothesized that PUE can alleviate the neurotoxicity effects of BPS by regulating oxidative stress and the BDNF/TrkB/CREB signaling pathway. To test this hypothesis, we exposed HT22 cells to different concentrations (40 µM and 80 µM) of BPS with or without 80 µM PUE. We analyzed the apoptosis rate, oxidative stress, the expression of proteins involved in the synaptic damage and the BDNF/TrkB/CREB pathway in order to assess the protective effect of PUE against BPS induced neurotoxicity effects in HT22 cells. We hope the findings will offer valuable insights to mitigate BPS-induced neural toxicity.

## 2. Material and Methods

### 2.1. Cell Culture and Treatment

HT22 cells were purchased from the Cell Bank of the Chinese Academy of Sciences (GNM47, China) and cultured in DMEM (C11995500BT, Gibco, USA) containing 10% FBS (04-001-1ACS, Biological Industries, Israel) and 1% penicillin-streptomycin (P1400, Solarbio, China) in a 5% CO_2_ atmosphere at 37 °C. HT22 cells were categorized into six experimental groups: control, PUE (80 µM PUE, Sarn Chemical Technology, Shanghai, China), BPS40 (40 µM BPS, Sigma Company, Shanghai, China), BPS80 (80 µM BPS), PUE + BPS40 (80 µM PUE + 40 µM BPS), and PUE + BPS80 (80 µM PUE + 80 µM BPS). For the PUE + BPS groups, cells were pretreated with PUE for 2 h, followed by BPS treated for 24 h. 

### 2.2. Cell Vitality Assay

The CCK-8 (BS350A, Biosharp, China) assay was used to assess the viability of HT22 cells treated with different concentrations of PUE and BPS. The cells (2 × 10^3^ cells per well) were seeded into 96-well plates and incubated for 24 h. And then treated with BPS and PUE at various concentrations for an additional 24 h. Subsequently, the cell culture medium was discarded, and 10 µL of CCK-8 solution was added to each well and incubated at 37 °C for 30 min to 1 h. Absorbance at 450 nm wavelength was measured using a full-wavelength scanning multifunctional microplate reader (Thermo Scientific Varioskan LUX, Waltham, MA, USA). Cell vitality was calculated according to the following formula:

$$\mathrm{Cell}\;\mathrm{vitality}=\frac{\mathrm{Absorbance}\;\mathrm{of}\;\mathrm{the}\;\mathrm{control}\;\mathrm{wells}-\mathrm{Absorbance}\;\mathrm{of}\;\mathrm{the}\;\mathrm{sample}\;\mathrm{wells}}{\mathrm{Absorbance}\;\mathrm{of}\;\mathrm{the}\;\mathrm{control}\;\mathrm{wells}-\mathrm{Absorbance}\;\mathrm{of}\;\mathrm{the}\;\mathrm{blank}\;\mathrm{wells}}\times 100\%$$

### 2.3. Reactive Oxygen Species Assay

Intracellular ROS levels in HT22 cells were measured using the dichlorodihydrofluorescein diacetate (DCFH-DA) method (CA1410, Solarbio, China). The HT22 cells were seeded into 6-well plates and incubated for 24 h. The cells were then treated with BPS and PUE at various concentrations for an additional 24 h. Subsequently, 1 mL of DCFH-DA solution was added to the cells, followed by incubation for 20 min at 37 °C. Following that, the cells were washed thrice with PBS (phosphate-buffered saline). The cells were observed using an fluorescence microscope (Olympus IX73, Janpan) with excitation and emission wavelengths of 488 and 525 nm, respectively, the levels of ROS were quantified using Image J software 1.53a (USA).

### 2.4. Determination of Oxidative Stress Index

Oxidative stress levels in HT22 cells were assessed using commercial kits for reduced glutathione by microplate method (A006-2-1, Nanjing, China), total SOD by WST-1 method (A001-3, Nanjing, China), and MDA by TBA method (A003-1, Nanjing, China). HT22 cells were treated with BPS at concentrations of 40 and 80 µM and PUE at 80 µM for 24 h. Subsequently, the cells were collected, and the absorbance values for GSH, SOD, and MDA were measured according to the manufacturer’s instructions.

### 2.5. Hoechst 33258 Staining

HT22 cells were seeded in a 6-well plate at a density of 1 × 10^5^/well for 24 h and then treated with BPS at concentrations of 40 and 80 µM and PUE at 80 µM for an additional 24 h. A 1 mL/well Hoechst 33258 (C1018, Beyotime, Shanghai, China) was added into each well after washed by PBS for three times. Fluorescence microscope (DM4B, Leica, Germany) was used to observe the morphological characteristics of nuclear to assess the occurrence of apoptosis.

### 2.6. Flow Cytometry Analysis

After treatment with different concentrations of BPS (40 and 80 µM) and PUE (80 µM) for 24 h, the cells were digested with trypsin ethylenediaminetetraacetic acid (EDTA)-free and resuspended with 300 µL of 1 × Binding Buffer containing 5 µL Annexin V-FITC and 5 µL PI. After washed by PBS for three times, the flow cytometry (BD, USA) was used to analyze the apoptosis rate of HT22 cells which treated by BPS or PUE. Flow J software 10.8.1 (BD, USA) was used to analyze the results.

### 2.7. Real-Time Polymerase Chain Reaction

HT22 cells were treated with BPS at concentrations of 40 and 80 µM and PUE at 80 µM for 24 h. Following treatment, cells were placed on ice, and RNA concentration and purity were assessed using a NanoDrop 2000 spectrometer (Thermo, Waltham, MA, USA). cDNA was synthesized using a reverse transcription kit (Monad, MR05101) and served as a template for qPCR. Following that, the mRNA expression levels of *BDNF*, *CREB*, *TrkB*, *PSD95*, *SYN1*, and *SYP* were quantified using a two-step fluorometer (Thermo, USA). The PCR conditions were as follows: pre-denaturation at 95 °C for 30 s, followed by 40 cycles of denaturation at 95 °C for 10 s, and annealing and elongation at 60 °C for 10 s. Relative gene expression levels were calculated using the 2^−ΔΔCt^ method. Primer sequences (Table 1) synthesized by Wuhan Jin Kairui Company (Wuhan, China).

### 2.8. Western Blotting (WB)

HT22 cells were treated with BPS (40 and 80 µM) and PUE (80 µM) for 24 h. Radioimmunoprecipitation assay buffer (R0010, Solarbio, Beijing, China) was used to extra total protein and the concentrations were determined using a Bicinchoninic acid (BCA) protein assay kit (P0010S, Beyotime, Shanghai, China). Proteins were separated via 12% sodium dodecyl sulfate-polyacrylamide gel electrophoresis (SDS-PAGE, G2042-4, servicebio, Wuhan, China) and transferred to a polyvinylidene fluoride (PVDF, IPVH00010, Servicebio, Wuhan, China) membrane. The membrane was blocked at room temperature for 1 h subsequently treated by primary antibodies such as BDNF (GB11559, 1:750, Servicebio, Wuhan, China), CREB (AF1018, 1:3000, Beyotime, Shanghai, China), TrkB (GB11295-1, 1:700, Servicebio, Wuhan, China), PSD95 (GB11277, 1:750, Servicebio, Wuhan, China), SYP (AF8091, 1:1500, Beyotime, Shanghai, China), SYN1 (K110156P, 1:3000, Solarbio, Beijing, China), Bax (GB114122, 1:600, Servicebio, Wuhan, China) and Bcl-2 (GB113375, 1:700, Servicebio, Wuhan, China) overnight at 4 °C. The following day, a goat anti-rabbit secondary antibody was added and incubated at room temperature on a shaker for 1 h. After washing thrice with tris buffered saline with tween-20 (TBST, T8220, Solarbio, Beijing, China), a chemiluminescence reagent and imaging system (ProteinSimplc, San Jose, CA, USA) was used to detect protein expression. Grayscale analysis was conducted with Image J software 1.53a.

### 2.9. Statistical Analysis

All experiments were conducted independently in triplicate. Data were statistically analyzed and mapped using the SPSS (version 28.0, SPSS Inc., Chicago, IL, USA) and presented as Mean ± Standard Deviation. Statistical significance was determined using analysis of variance followed by the least significant difference test. A *p* < 0.05 was considered statistically significant.

## 3. Results

### 3.1. Effects of Bisphenol S and Puerarin on HT22 Cell Viability

**Figure 2:**
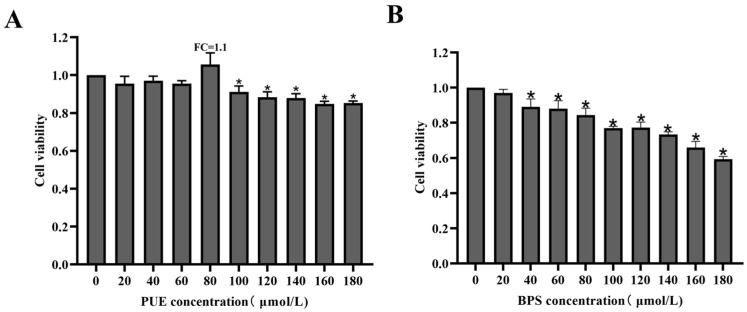
Cell viability of HT22 cells after treated by PUE and BPS. (**A**) PUE (**B**) BPS. Data are expressed as $\bar{X}$ ± SD; *: *p* < 0.05, compared to control group.

The CCK-8 results showed that no significant decrease in cell viability was observed when HT22 cells were exposed to BPS concentrations < 40 µM for 24 h. However, at concentrations > 40 µM, BPS significantly reduced the HT22 cell viability (*p* < 0.05). Similarly, high concentration PUE also decreased the cell viability of HT22 cell. However, the concentrations of PUE at 80 µM increased the cell viability (*p* > 0.05, FC = 1.1) (Figure 2A). Therefore, we selected 40 µM as the minimum BPS concentration that significantly affected HT22 cell viability and 80 µM as the maximum concentration at which BPS causes neurotoxic effects and obviously cell damage. Thus, 80 µM PUE was selected as the optimal concentration to complete the next experiment, as the PUE in 80 µM did not affect cell viability and exhibited protective effects.

### 3.2. Puerarin Alleviates Bisphenol S-Induced Oxidative Stress in HT22 Cells

**Figure 3:**
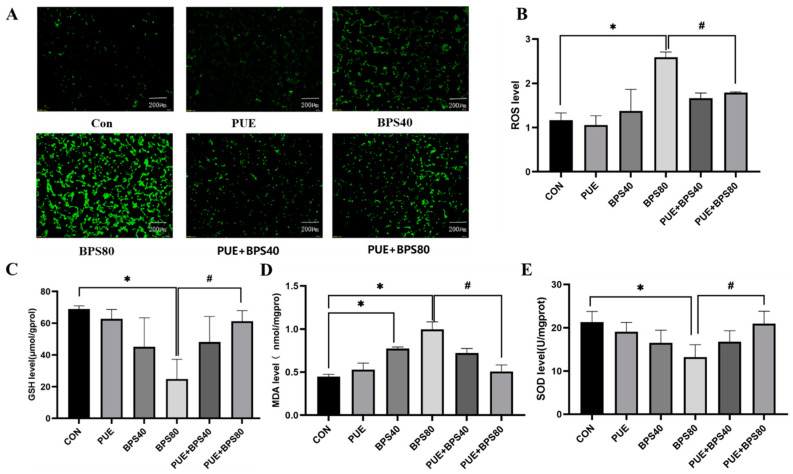
BPS induces oxidative stress in HT22 cells. All data are expressed as X ± SD, *n* = 3. (**A**) Microscopic image showing ROS detection, visualized via green fluorescence intensity using the DCFH-DA probe; (**B**) quantification of ROS using Image J; (**C**–**E**) effect of BPS on GSH, MDA, and SOD in HT22 cells. *: *p* < 0.05, BPS80 group compared to control group; #: *p* < 0.05 BPS80 group compared to the PUE + BPS group.

The ROS analysis results showed that the BPS-treated cells experienced a significant increase in ROS levels compared to those in the control group. However, PUE treatment significantly ameliorated this increase, with significant protective effects observed in the BPS80 and PUE + BPS80 groups (Figure 3A,B, *p* < 0.05). Figure 3C–E shows that BPS treatment for 24 h significantly reduced GSH levels and SOD activity in HT22 cells compared to in the control group (*p* < 0.05), while MDA levels were significantly increased (*p* < 0.05). However, but PUE pretreatment significantly reduced the production of MDA (Figure 3D, *p* < 0.05) and reduced GSH levels and SOD activity in HT22 cells (Figure 3C,E, *p* < 0.05). These results demonstrate that PUE alleviates BPS-induced oxidative stress in HT22 cells.

### 3.3. Puerarin Ameliorates Bisphenol S-Induced Apoptosis in HT22 Cells

**Figure 4:**
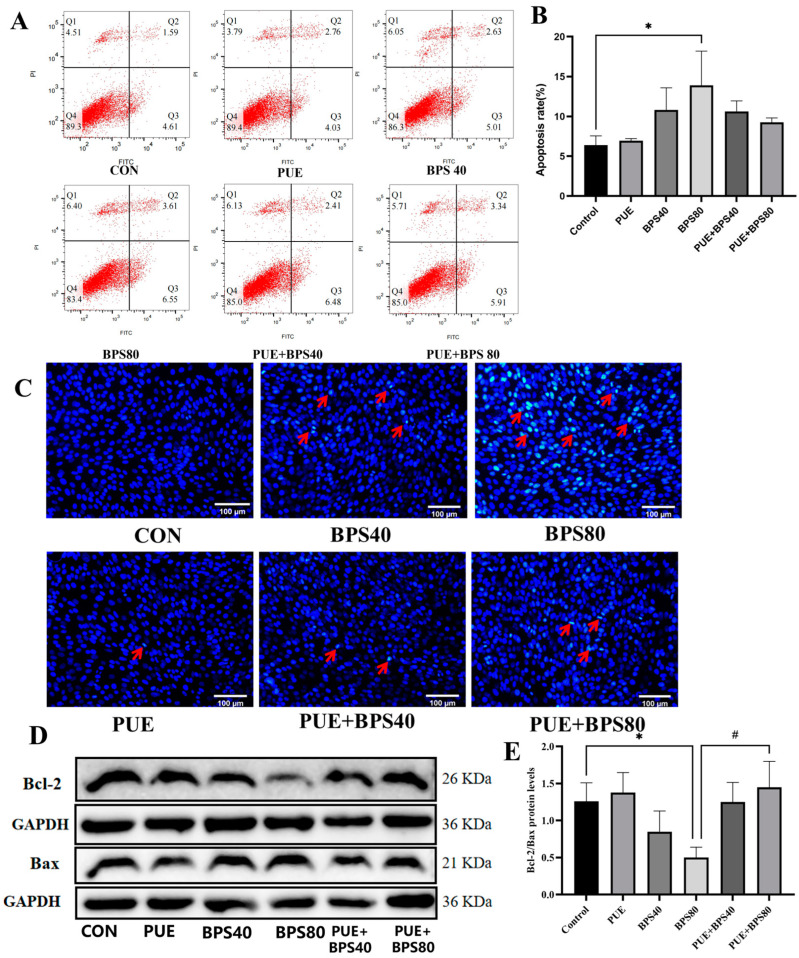
Puerarin ameliorates Bisphenol S-induced apoptosis in HT22 cells. (**A**) Flow cytometry dot plot of HT22 cells after 24 h of PUE intervention and BPS treatment (red arrows represent apoptotic cells); (**B**) Quantification of apoptosis rate of each group via FLOW JO. (**C**) Observation of apoptosis in HT22 cells via Hoechst 33258 staining, red arrows highlighting apoptotic cells; (**D**) Apoptosis-related protein expression (Bax and Bcl-2); (**E**) Quantitative results of apoptosis-related proteins. *: *p* < 0.05, BPS80 group compared to control group; #: *p* < 0.05, BPS80 group compared to the PUE + BPS group.

According to the result of flow cytometry results, the apoptosis rate of HT22 cells in the BPS80 group significantly increased compared with the control group (Figure 4B, *p* < 0.05). Although the apoptosis rate of HT22 cells showed a slight increase following PUE intervention, the change was not statistically significant (Figure 4B, *p* > 0.05). Additionally, Hoechst 33258 staining and WB analysis of apoptosis inhibitory protein Bcl-2 and pro-apoptotic protein Bax were conducted to further evaluate the apoptotic cells. The Hoechst 33258 staining results showed that exposure to BPS for 24 h could significantly increase the apoptosis rate of HT22 cells (Figure 4C, *p* < 0.05). However, PUE pretreatment alleviated the apoptosis rate of HT22 cells induced by BPS. Similarly, WB results showed that BPS80 decreased the ratio of Bcl-2/Bax compared with control groups. However, after 24 h of PUE intervention, the Bcl-2/Bax ratio in the PUE + BPS80 group was significantly higher than that of the BPS80 group (Figure 4E, *p* < 0.05). These results indicates that PUE exerts a remissive effect on BPS-induced apoptosis in the HT22 cells.

### 3.4. Puerarin Alleviates Bisphenol S-Downregulated Synaptic Associated Protein in HT22 Cells

**Figure 5:**
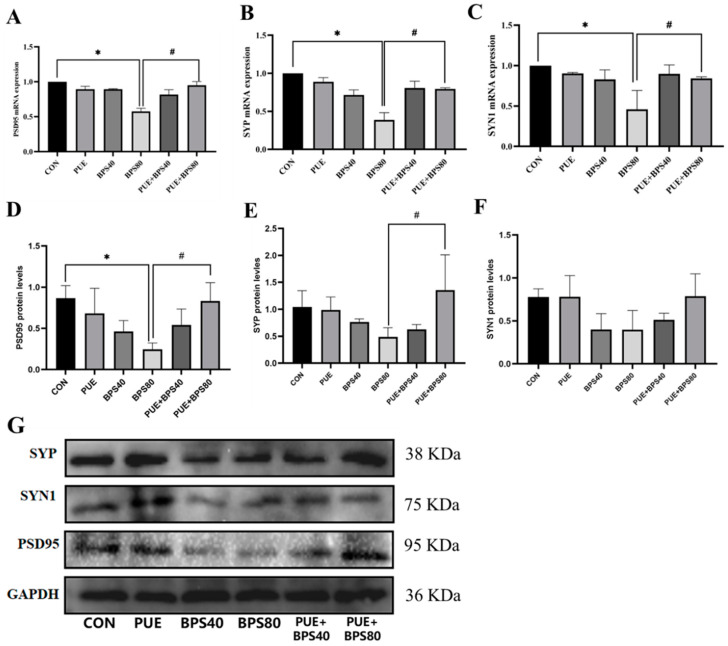
PUE reduces BPS effect on synaptic plasticity in HT22 cells. Data were expressed as X ± SD, n = 3. (**A**–**C**) mRNA levels of PSD95, SYP, and SYN1. (**D**–**F**) protein levels of PSD95, SYP, and SYN1, respectively. (**G**) Representative Western blot of PSD95, SYP, and SYN1. *: *p* < 0.05 between the BPS treatment group and the control group; #: *p* < 0.05 compared to the PUE + BPS group.

To determine the effect of PUE in BPS-mediated synaptic injury in HT22 cells, we assessed the mRNA and proteins expression levels of synaptic plasticity relative genes including PSD95, SYP, and SYN1. The RT-PCR results showed that the mRNA expressions of PSD95, SYP, and SYN1 were significantly downregulated in the BPS80 group compared with the control group. After 24 h of PUE intervention, the mRNA levels were significantly increased in the PUE + BPS80 group compared with BPS80 group (Figure 5A–C, *p* < 0.05). The WB results also showed a significant decrease in PSD95 protein expression 24 h after BPS80 treatment, which was alleviated following 24 h of PUE intervention (Figure 5D, *p* < 0.05). SYN1 and SYP protein expressions showed a downward trend in the treatment group compared with the control group (Figure 5E,F, *p* > 0.05) and SYP protein expression was significantly increased following PUE intervention (*p* < 0.05).

### 3.5. Puerarin Alleviates Bisphenol S-Downregulated BDNF, CREB, and TrkB in HT22 Cells

**Figure 6:**
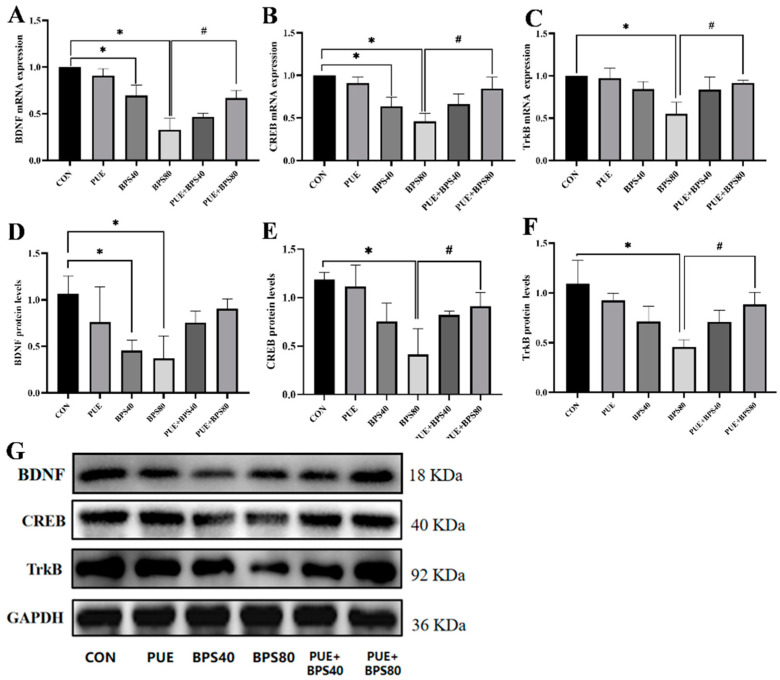
BPS and PUE effect on BDNF-TrkB-CREB signaling pathway. Data are expressed as X ± SD, n = 3. (**A**–**C**) mRNA levels of BDNF, CREB and TrkB. (**D**–**F**) protein levels of BDNF, CREB and TrkB, respectively. (**G**) Representative Western blot of BDNF, CREB and TrkB. *: *p* < 0.05 between the BPS treatment group and the control group; #: *p* < 0.05 compared to the PUE + BPS group.

Additionally, we measured the mRNA and protein levels of BDNF, CREB, and TrkB. The results showed that the mRNA levels of BDNF and CREB were significantly reduced after 24 h of BPS treatment in HT22 cells compared with the control group. Conversely, the TrkB gene expression levels were only significantly reduced only in the BPS80 group. After 24 h of PUE intervention, the mRNA levels were significantly increased in the PUE + BPS80 group compared with the BPS80 group (Figure 6A–C, *p* < 0.05). Meanwhile, BPS exposure significantly downregulated BDNF protein levels (Figure 6D, *p* < 0.05), CREB and TrkB protein expressions levels in the BPS80 group were significantly lower than in the control group (Figure 6E,F, *p* < 0.05). However, these protein expressions levels were significantly improved after 24 h of PUE treatment (Figure 6D–F, *p* < 0.05).

## 4. Discussion

BPS is considered the most common alternative to BPA [21], and the toxic effects of BPS have received increasing attention. The majority of studies have elucidated that the BPS can promote the development of cancer [22], and can induce toxicity, including hepatotoxicity [23], transgenerational toxicity and negative effects on reproduction [24], especially neurotoxicity [25]. However, effective interventions for controlling BPS-induced toxicity remain limited. In this study, our aims were to examine the protective effect of PUE for against BPS-induced neurotoxicity and related mechanisms in order to provide strategies for preventing and reducing the damage caused by BPS.

Recent studies have shown that BPS plays an important role in promoting apoptosis. He et al. found that exposure to BPS for 48 h can increase the apoptosis rate and proapoptotic Bax protein levels in SK–N–SH cells, leading to cell death, and BPS can also induce apoptosis in neural stem cells and cause neurotoxicity in rats [12]. In the present study, we demonstrated that BPS induced increased apoptosis in HT22 cells, as confirmed via Hoechst 33258 staining and flow cytometry; decreased the protein and mRNA expression levels of Bcl-2; and increased the protein and mRNA expression levels of Bax. PUE is a class of phytoestrogens that significantly improve nerve function [26]. It has been shown to play a neuroprotective role by inhibiting cell apoptosis. A study has reported that PUE can alleviate the apoptosis of hippocampal cells in ischemic stroke [20]. In addition to its anti-apoptotic effects, puerarin also demonstrates antioxidant activity. A previous study indicated that puerarin could improve oxidative damage and memory impairment in rats by reversing the alterations in the levels of GSH, SOD, and MDA in the hippocampus induced by cycloheximide (CXM) [27]. In addition, an increasing number of studies have confirmed that puerarin can improve cognitive dysfunction in Alzheimer’s disease (AD) by alleviating oxidative stress [28]. Similarly, our results also showed that PUE reversed BPS-induced apoptosis and oxidative damage in HT22 cells, with significant improvements observed in apoptosis-related protein expression.

The findings of experiments in vivo also indicated that BPS can cause oxidative stress in the brains of adult zebrafish, thereby inducing anxiety and fear responses [29]. Previous studies have shown that BPS can increase ROS levels and induce apoptosis [30,31]. Additionally, BPS induces neurotoxicity in IMR-32 cells by increasing MDA content, reducing SOD activity, and producing excessive ROS [10,32]; studies have shown that PUE treatment can increase SOD activity, alleviate oxidative stress, and significantly inhibit ROS production in mice with subarachnoid hemorrhage (SAH) [26]. A pharmacological study on PUE confirmed its neuroprotective effects, showing that PUE can alleviate neurological dysfunction in rats after SAH by increasing SOD and GSH levels while reducing MDA and glutathione oxidized levels [33]. Similarly, our findings showed that BPS increases ROS production while decreasing GSH and SOD levels in HT22 cells. but PUE intervention effectively inhibited the increase in ROS production and altered GSH, MDA, and SOD levels in HT22 cells induced by BPS, thereby decreasing BPS-induced oxidative stress.

It is reported that BPS can induce axonal damage and remodeling [34],inhibits neuronal maturation, causes neuronal myelin degeneration, changes the number of hippocampal synapses and damaged the learning and memory functions [12]. An in vitro study assessing the neurotoxicity of BPS revealed that BPS may exert neurotoxicity by reducing neurite length [35]. The BDNF/TrkB/CREB signaling pathway regulates synaptic plasticity, and plays a significant role in learning and memory functions. This BDNF/TrkB/CREB signaling pathway can also regulate neurotrophins and hippocampal synapsis-related genes to reduce brain injury in mice [36]. Our previous study has also confirmed that BPS may exert neurotoxicity on the mouse hippocampus by regulating BDNF/TrkB/CREB pathways [9]. Meanwhile, the inhibition of the BDNF/TrkB/CREB pathway can increase the apoptosis rate of neuron [37]. CREB is essential for maintaining synaptic plasticity and for the transcription of genes encoding synapse-associated proteins, such as PSD95 [38]. In this study, we demonstrated that exposure to BPS decreased the levels of mRNA and protein of BDNF, CREB, and TrkB in HT22 cells and downregulated the levels of synapse-related genes, including PSD95, SYN1, and SYP. Following PUE intervention, the BDNF, CREB and TrkB gene expression levels increased, and synapse-related genes (PSD95, SYN1, and SYP) expression levels were also upregulated in HT22 cells. In general, our findings demonstrated that PUE has the potential to protect against the neurotoxicity caused by BPS by targeting the oxidative stress and the BDNF/TrkB/CREB signaling pathway to reduce apoptosis.

## 5. Conclusions

Exposure to BPS leads to the apoptosis rate increase, but this effect is reversed by PUE. PUE also relieves the oxidative damage induced by BPS. BPS inhibited the expression of PSD95, SYN1, SYP and BDNF/TrkB/CREB signaling pathway, but PUE intervention could alleviate the adverse effect induced by BPS. In conclusion, this study demonstrated the protective effect of PUE which alleviate the neurotoxicity effects of BPS by regulating oxidative stress, synaptic damage, and the BDNF/TrkB/CREB pathway. However, the neuroprotective effect of PUE and its mechanism need to be further verified in vivo and in vitro. Importantly, this study provides a new perspective for the intervention of neurotoxic effects induced by BPS, and also provides theoretical support for the subsequent study of neuroprotective effects of PUE.

## Figures and Tables

**Table 1:** Primer sequences for neutrally associated genes.

Genes	Primer	Sequences (5′–3′)
*BDNF*	Forward	GCCCATGAAAGAAGTAAACGTCC
	Reverse	AGTGTCAGCCAGTGATGTCGTC
*CREB*	Forward	GGAGCAGACAACAGCAGAGTG
	Reverse	GGCATGGATACCTGGGCTAATGTG
*TrkB*	Forward	CGCTTCAGTGGTTCTACA
	Reverse	CCTTCCCATACTCGTTCTT
*PSD95*	Forward	TCACATTGGAAAGGGGTAA
	Reverse	AAGATGGATGGGTCGTCA
*SYP*	Forward	CTTCGGCGACTTCTACTACTTT
	Reverse	GGAGCGGATGGATGTTTG
*SYN1*	Forward	CTTCTCGTCGCTGTCTAA
	Reverse	ATGGATCTTCTTCCCTTT
*GAPDH*	Forward	CCTCGTCCC GTAGACAAAATG
	Reverse	TGAGGTCAATGAAGG GGTCGT

## Data Availability

Data are available from the corresponding author by request.

## References

[1] Wang J., Hong X., Liu W., Zhang L., Yan S., Li Z., Zha J. (2024). Comprehensive assessment of the safety of bisphenol A and its analogs based on multi-toxicity tests in vitro. J. Hazard. Mater..

[2] Liao C., Liu F., Guo Y., Moon H.-B., Nakata H., Wu Q., Kannan K. (2012). Occurrence of eight bisphenol analogues in indoor dust from the United States and several Asian countries: Implications for human exposure. Environ. Sci. Technol..

[3] Wu L.-H., Zhang X.-M., Wang F., Gao C.-J., Chen D., Palumbo J.R., Guo Y., Zeng E.Y. (2018). Occurrence of bisphenol S in the environment and implications for human exposure: A short review. Sci. Total Environ..

[4] Zhang H., Zhang Y., Li J., Yang M. (2019). Occurrence and exposure assessment of bisphenol analogues in source water and drinking water in China. Sci. Total Environ..

[5] Chen M.Y., Ike M., Fujita M. (2002). Acute toxicity, mutagenicity, and estrogenicity of bisphenol-A and other bisphenols. Environ. Toxicol..

[6] Jiang Y., Li J., Xu S., Zhou Y., Zhao H., Li Y., Xiong C., Sun X., Liu H., Liu W. (2020). Prenatal exposure to bisphenol A and its alternatives and child neurodevelopment at 2 years. J. Hazard. Mater..

[7] Wang Y.-X., Dai W., Li Y.-Z., Wu Z.-Y., Kan Y.-Q., Zeng H.-C., He Q.-Z. (2023). Bisphenol S induces oxidative stress-mediated impairment of testosterone synthesis by inhibiting the Nrf2/HO-1 signaling pathway. J. Biochem. Mol. Toxicol..

[8] Wu Z.-Y., Luo L., Kan Y.-Q., Qin M.-L., Li H.-T., He Q.-Z., Zeng H.-C. (2023). Puerarin Prevents Bisphenol S Induced Lipid Accumulation by Reducing Liver Lipid Synthesis and Promoting Lipid Metabolism in C57BL/6J Mice. Toxics.

[9] Li Y.-Z., Wu Z.-Y., Zhu B.-Q., Wang Y.-X., Kan Y.-Q., Zeng H.-C. (2022). The BDNF-TrkB-CREB Signalling Pathway Is Involved in Bisphenol S-Induced Neurotoxicity in Male Mice by Regulating Methylation. Toxics.

[10] Wang C., He J., Xu T., Han H., Zhu Z., Meng L., Pang Q., Fan R. (2021). Bisphenol A(BPA), BPS and BPB-induced oxidative stress and apoptosis mediated by mitochondria in human neuroblastoma cell lines. Ecotoxicol. Environ. Saf..

[11] Harnett K.G., Chin A., Schuh S.M. (2021). BPA and BPA alternatives BPS, BPAF, and TMBPF, induce cytotoxicity and apoptosis in rat and human stem cells. Ecotoxicol. Environ. Saf..

[12] Tiwari S., Phoolmala, Goyal S., Yadav R.K., Chaturvedi R.K. (2024). Bisphenol-F and Bisphenol-S (BPF and BPS) Impair the Stemness of Neural Stem Cells and Neuronal Fate Decision in the Hippocampus Leading to Cognitive Dysfunctions. Mol. Neurobiol..

[13] Guo C., Liu Y., Fang M.-S., Li Y., Li W., Mahaman Y.A.R., Zeng K., Xia Y., Ke D., Liu R. (2020). ω-3PUFAs Improve Cognitive Impairments Through Ser133 Phosphorylation of CREB Upregulating BDNF/TrkB Signal in Schizophrenia. Neurotherapeutics.

[14] Yang P., Chen H., Wang T., Su H., Li J., He Y., Su S. (2023). Electroacupuncture promotes synaptic plasticity in rats with chronic inflammatory pain-related depression by upregulating BDNF/TrkB/CREB signaling pathway. Brain Behav..

[15] Li R., Yang W., Yan X., Zhou X., Song X., Liu C., Zhang Y., Li J. (2024). Folic acid mitigates the developmental and neurotoxic effects of bisphenol A in zebrafish by inhibiting the oxidative stress/JNK signaling pathway. Ecotoxicol. Environ. Saf..

[16] Zhou Y.-X., Zhang H., Peng C. (2014). Puerarin: A review of pharmacological effects. Phytother. Res..

[17] Lai Y., Yang N., Shi D., Ma X., Huang Y., Lu J., Zhang X., Zhou H., Gao W., Mao C. (2024). Puerarin enhances TFEB-mediated autophagy and attenuates ROS-induced pyroptosis after ischemic injury of random-pattern skin flaps. Eur. J. Pharmacol..

[18] He L., Wu X., Zhang X., Li X., Lin X., Huang Y., Wu J. (2022). Puerarin protects against H_2_O_2_-induced apoptosis of HTR-8/SVneo cells by regulating the miR-20a-5p/VEGFA/Akt axis. Placenta.

[19] Liu Y., Hu Z., Wang J., Liao Y., Shu L. (2023). Puerarin alleviates depressive-like behaviors in high-fat diet-induced diabetic mice via modulating hippocampal GLP-1R/BDNF/TrkB signaling. Nutr. Neurosci..

[20] Zhang Q., Yao M., Qi J., Song R., Wang L., Li J., Zhou X., Chang D., Huang Q., Li L. (2023). Puerarin inhibited oxidative stress and alleviated cerebral ischemia-reperfusion injury through PI3K/Akt/Nrf2 signaling pathway. Front. Pharmacol..

[21] Naderi M., Kwong R.W.M. (2020). A comprehensive review of the neurobehavioral effects of bisphenol S and the mechanisms of action: New insights from in vitro and in vivo models. Environ. Int..

[22] Yuan X., Chen K., Zheng F., Xu S., Li Y., Wang Y., Ni H., Wang F., Cui Z., Qin Y. (2023). Low-dose BPA and its substitute BPS promote ovarian cancer cell stemness via a non-canonical PINK1/p53 mitophagic signaling. J. Hazard. Mater..

[23] Liang J., Xu C., Xu J., Yang C., Kong W., Xiao Z., Chen X., Liu Q., Weng Z., Wang J. (2023). PPARα Senses Bisphenol S to Trigger EP300-Mediated Autophagy Blockage and Hepatic Steatosis. Environ. Sci. Technol..

[24] Zhao M., Xie Y., Xu X., Zhang Z., Shen C., Chen X., Zhu B., Yang L., Zhou B. (2024). Reproductive and transgenerational toxicity of bisphenol S exposure in pregnant rats: Insights into hormonal imbalance and steroid biosynthesis pathway disruption. Sci. Total Environ..

[25] Meng L., Gui S., Ouyang Z., Wu Y., Zhuang Y., Pang Q., Fan R. (2023). Low-dose bisphenols exposure sex-specifically induces neurodevelopmental toxicity in juvenile rats and the antagonism of EGCG. J. Hazard. Mater..

[26] Zhang Y., Yang X., Ge X., Zhang F. (2019). Puerarin attenuates neurological deficits via Bcl-2/Bax/cleaved caspase-3 and Sirt3/SOD2 apoptotic pathways in subarachnoid hemorrhage mice. Biomed. Pharmacother..

[27] Wu K.-J., Lien J.-C., Wu C.-R. (2024). Puerarin Attenuates Cycloheximide-Induced Oxidative Damage and Memory-Consolidation Impairment in Rats. J. Integr. Neurosci..

[28] Zhou Y., Xie N., Li L., Zou Y., Zhang X., Dong M. (2014). Puerarin alleviates cognitive impairment and oxidative stress in APP/PS1 transgenic mice. Int. J. Neuropsychopharmacol..

[29] Salahinejad A., Attaran A., Naderi M., Meuthen D., Niyogi S., Chivers D.P. (2021). Chronic exposure to bisphenol S induces oxidative stress, abnormal anxiety, and fear responses in adult zebrafish (Danio rerio). Sci. Total Environ..

[30] Cheung E.C., Vousden K.H. (2022). The role of ROS in tumour development and progression. Nat. Rev. Cancer.

[31] He Q.-Z., Zhu B.-Q., Xu X.-N., Zeng H.-C. (2021). Role of the BDNF/TrkB/CREB signaling pathway in the cytotoxicity of bisphenol S in SK-N-SH cells. J. Biochem. Mol. Toxicol..

[32] Wang L., Huang C., Li L., Pang Q., Wang C., Fan R. (2023). In vitro and in silico assessment of GPER-dependent neurocytotoxicity of emerging bisphenols. Sci. Total Environ..

[33] Huang Y., Wu H., Hu Y., Zhou C., Wu J., Wu Y., Wang H., Lenahan C., Huang L., Nie S. (2022). Puerarin Attenuates Oxidative Stress and Ferroptosis via AMPK/PGC1α/Nrf2 Pathway after Subarachnoid Hemorrhage in Rats. Antioxidants.

[34] Hu F., Li T., Gong H., Chen Z., Jin Y., Xu G., Wang M. (2017). Bisphenol A Impairs Synaptic Plasticity by Both Pre- and Postsynaptic Mechanisms. Adv. Sci..

[35] Liang X., Yin N., Liang S., Yang R., Liu S., Lu Y., Jiang L., Zhou Q., Jiang G., Faiola F. (2020). Bisphenol A and several derivatives exert neural toxicity in human neuron-like cells by decreasing neurite length. Food Chem. Toxicol..

[36] Bai Y., Han Q., Dong B., Lin H., Jiang Y., Zhang X., Chen H., Yu Y. (2022). PPARα contributes to the therapeutic effect of hydrogen gas against sepsis-associated encephalopathy with the regulation to the CREB-BDNF signaling pathway and hippocampal neuron plasticity-related gene expression. Brain Res. Bull..

[37] Li N., Liu G.T. (2010). The novel squamosamide derivative FLZ enhances BDNF/TrkB/CREB signaling and inhibits neuronal apoptosis in APP/PS1 mice. Acta Pharmacol. Sin..

[38] Wang C.S., Kavalali E.T., Monteggia L.M. (2022). BDNF signaling in context: From synaptic regulation to psychiatric disorders. Cell.

